# Phenotypic characterization and epidemiology of extended-spectrum *β*-lactamase-producing *Enterobacteriaceae* strains from urinary tract infections in Garoua, Cameroon

**DOI:** 10.3389/fpubh.2023.1187934

**Published:** 2023-06-29

**Authors:** Karyom Djim-Adjim-Ngana, Brunel W. Mbiakop, Leila A. Oumar, Hermann L. Munshili Njifon, Cedric Tchinda Fossi, Elisee L. Embolo Enyegue, Mohamed M. Mouiche Mouliom, Siméon P. Fodouop Chegaing, Louis Deweerdt, Nicolas Njintang Yanou, Julien A. Nguinkal

**Affiliations:** ^1^Centre for Research on Health and Priority Pathologies, Institute of Medical Research and Medicinal Plant Studies, Yaounde, Cameroon; ^2^Department of Veterinary Public Health, School of Veterinary Medicine and Sciences, University of Ngaoundere, Ngaoundere, Cameroon; ^3^Centre Pasteur of Cameroon Annex of Garoua, Garoua, Cameroon; ^4^Department of Biomedical Sciences, University of Ngaoundere, Ngaoundere, Cameroon; ^5^Centre for Research on Medicinal Plants and Traditional Medicine, Institute of Medical Research and Medicinal Plant Studies, Yaounde, Cameroon; ^6^Department of Infectious Disease Epidemiology, Bernhard Nocht Institute for Tropical Medicine, Hamburg, Germany

**Keywords:** antibiotics, double synergy test, ESBL, UTI—Urinary tract infection, antibiotics, *Enterobacteriaceae*, multidrug resistance, microbial susceptibility

## Abstract

**Background and objectives:**

The emergence of extended-spectrum beta-lactamase-producing *Enterobacteriaceae* (ESBL-E) is causing increased morbidity and mortality around the world as a result of therapeutic failures. ESBL-E are priority pathogens due to their multidrug resistance (MDR). In Northern Cameroon, ESBL-producing bacteria, particularly in urinary tract infections (UTIs), are being increasingly isolated. This study aimed to retrospectively determine the prevalence of multi-drug resistant ESBL strains isolated from UTIs in Northern Cameroon and to evaluate the effectiveness of the ATB UR Gallery of BioMérieux in diagnosing ESBL-E in clinical settings.

**Methods:**

Standard microbiology protocols and statistical tools were utilized to identify ESBL-producing bacteria and characterize their phenotypic susceptibility and resistance profiles in the study population.

**Results:**

Out of the 144 enterobacteria isolates successfully cultured, 59 (41%) were identified as MDR strains. The ATB UR EU gallery identified 33 (23%) multi-drug resistant ESBL-producing strains, while the double synergy test identified 35 strains without disc reconciliation and 38 strains after reconciliation. The most prevalent ESBL-E isolate was Escherichia coli, accounting for 77.1% of the isolates, followed by *Klebsiella pneumoniae* (20%) and *Enterobacter aerogenes* (2.9%). Additionally, the study revealed the emergence of Imipenem resistance (5.7%), a critical last-resort antibiotic. However, all ESBL strains were sensitive to Fosfomycin (FSF/FOS), demonstrating its potential as an effective therapeutic option. Moreover, 37% of the ESBL producers exhibited co-resistance to over 20 different antibiotics.

**Conclusion:**

This study provides valuable insights into the prevalence and susceptibility patterns of ESBL-E associated with UTIs in Northern Cameroon. These insights emphasizes the importance of implementing appropriate treatment guidelines and antimicrobial stewardship measures to mitigate the spread and impact of MDR ESBL-producing strains on public health.

## 1. Introduction

Urinary tract infections (UTIs) are among the most common bacterial infections affecting subjects of all ages. To avoid complications, UTIs necessitate a particular medical attention, including antimicrobial therapy, which eventually leads to the selection of multi-resistant strains in both hospital and community settings (1, 2). Most UTIs are caused by *Enterobacteriaceae*, a large family of Gram-negative bacteria that includes several pathogens such as *Klebsiella* sp., *Enterobacter* sp., *Citrobacter* sp., *Salmonella* sp., *Escherichia coli, Shigella* sp., and others. Some Enterobacterales can produce hydrolytic enzymes called extended-spectrum beta-lactamases, which inactivate and destroy most of the commonly used extended-spectrum antibiotics, including penicillins, third- and fourth-generation cephalosporins, and monobactams, making these drugs ineffective for treating ESBL-associated bacterial infections (3, 4). Due to their remarkable safety profiles and broad-spectrum activity against a broad range of pathogens, beta-lactam antibiotics remain a preferred choice for first-line care (5). However, ESBL-producing bacteria can hydrolyze (inactivate) most beta-lactams except cephamycins and carbacephems (6). This phenomenon represents Enterobacteriaceae's primary drugs resistance mechanism (3, 7, 8).

The prevalence of extended-spectrum beta-lactamase-producing *Enterobacteriaceae* (ESBL-E) is rapidly increasing globally (9–11), and ESBL-E are recognized as one of the most emerging multidrug-resistant bacteria (12–14). The main drivers underlying this rapid spread of antimicrobial resistance (AMR) and, thus, the increasing prevalence of multidrug-resistant ESBL-producing bacteria (MDR-ESBL) around the world are plasmid-mediated transmission of ESBL coding genes and horizontal gene transfer (15–17). Unfortunately, this increased incidence is often associated with multi-drug resistance (MDR) to the majority of front-line antibiotic families, severely limiting the therapeutic options available. MDR-ESBL has become a public health emergency (1, 18), particularly in developing countries with limited resources such as Cameroon, where the prevalence of ESBL-E is alarmingly high, with some studies reporting prevalences of up to 49% (19, 20). These MDR pathogens are known to cause nosocomial and community infections (19, 21–24), particularly in the intensive-care unit (ICU). The majority of MDR-ESBL producers are isolated from critical care patients (25, 26), emphasizing the urgent need for informed AMR surveillance and stewardship.

Despite the lack of a national MDR-ESBL monitoring program in the country, there has been so far a few data published on the subject in order to determine the scope of this phenomenon. Gangoué-Piéboji et al. conducted the groundbreaking ESBL study, which focused on *K. pneumoniae* and *E. coli* strains (21). Lonchel et al. supplemented this with a second study in the Ngaoundéré region, which revealed a 16% prevalence of ESBL-E in the community (22). At the same time, Djuikoue et al. reported that all ESBL-producing *E. coli* strains contained CTX-M enzymes from group I (27). In 2017, a study of 86 *E. coli* isolates from women in Yaoundé found that 45.3% of ESBL-producing strains were all CTX-M type, with previous antibiotics therapy as risk factors (28). More recently, Djim-Adjim-Ngana et.al. found a 19.3% prevalence of ESBL-E in urinary tract infections in children with prior hospitalization and recent antibiotics use as the main risk factors (19). As we know today, the severity of UTIs, as well as the increased morbidity and mortality, have been associated with multidrug-resistance of ESBL-E (29–31). Identifying patients at risk of ESBL remains a significant challenge to optimize antibiotic therapy and improve patient prognosis (32).

A serious public health concern in the country is to reach a therapeutic impasse in which no antibiotic will be effective against such infections, as the co-occurrence of ESBL-positive isolates and AMR is now considered a national public health threat. The time-consuming nature of the laboratory diagnoses in the country, as well as the overuse and inappropriate antimicrobial therapy, may exacerbate this concern as in other resources-limited countries. Tools such as ATB UR EU Gallery can be used to detect ESBL-producing bacteria in clinical and public health laboratories for proper laboratory surveillance of AMR. This study was therefore carried out in an effort to provide phenotypic characteristics and epidemiological relevant data for MDR-ESBL surveillance and stewardship with the help of two standard phenotypic methods (ATB UR EU Gallery and Double synergy test).

## 2. Materials and methods

### 2.1. Study design

The research was carried out in Garoua, the capital of the North Cameroon region, which lies between the Adamaoua and Far-North regions of the country. It shares borders with Nigeria in the west and the Central African Republic in the east. This is a cross-sectional, retrospective study to analyze multidrug-resistant enterobacteria strains that were characterized in the Centre Pasteur du Cameroun Annexe bacteriology lab in Garoua (CPCAG) in 2017. We collected urine samples from subjects with suspected urinary tract infections (UTIs) attending health facilities in Garoua. The subjects' ages, sex and recent history of antibiotics therapy were documented.

### 2.2. Laboratory analyses

Urine was collected for diagnostic purposes using sterile urine collection bags (Urinocol^®^, B. Braun Medical, France) according to the manufacturer's instructions. Each urine specimen was subjected to a routine urinary cytobacteriological examination that included: (i) a count of leukocytes and red blood cells in Kova^®^ cells, which also allowed for the recording of other elements such as epithelial cells, cylinders, crystals, and so on; and (ii) a bacterial cells culture with a count of germs (bacteriuria). A urinary tract infection was characterized by leukocyturia (>104/ml leukocytes) and bacteriuria [>105 colony-forming units (CFU)/ml]. The laboratory analysis revealed 144 *Enterobacteriaceae* strains that were associated with UTIs. In this study, we analyzed 59 strains of enterobacteria that were MDR (Supplementary File 1). The French Microbiology Society's Antibiotic Committee defined these strains as being resistant to third- and/or fourth-generation cephalosporins (CA-SFM, 2019). We first utilized chromogenic medium to culture and isolate *Enterobacteria*. The standard API 20E gallery (Bio Mérieux SA, France) and BioRad Uriselect^®^ were used to confirm the species identities of the isolated bacterial strains (Supplementary Figure 3).

### 2.3. Study of the sensitivity of bacterial strains to different antibiotics

The ATB UR gallery was used to test the antibiotic resistance of *Enterobacteriaceae* (33). The ATB^TM^ UR EU (08) gallery is a qualitative standardized method for determining the antibiotic susceptibility of urinary Enterobacterales in a semi-solid medium. It is very comparable to the reference methods, such as agar dilution or microdilution. There are several pairs of cups in the ATB UR EU gallery (08). The first pair acts as a growth control without the use of antibiotics. The remaining 15 have one or two antibiotic concentrations (c and C). The bacteria to be tested are suspended, mixed with culture medium, and injected into the gallery. Following incubation, the growth is visually analyzed with an ATB, mini API^®^ machine, or both. The result allows the strain to be classified as Susceptible, Intermediate, or Resistant. Note that, as of 2020, the EUCAST committee refers to the sensitive and intermediate categories as “Standard Dose Sensitive” and “High Dose Sensitive,” respectively. The “Resistant” category has remained unchanged. In total, we tested 25 antibacterial drugs commonly prescribed in the country, including six classes: Beta-lactams (*n* = 13), Aminoglycosides (*n* = 3), Quinolones (*n* = 6), Sulfonamides (*n* = 1), Nitrofurans (*n* = 1), and Fosfomycin (*n* = 1).

### 2.4. Detection of extended spectrum beta-lactamase producers

ESBL is suspected when the inhibition diameter of third and/or fourth generation cephalosporins and/or monobactam decreases. All tests were performed in accordance with the Antibiotic Committee of the French Society of Microbiology's recommendations (CA-SFM 2019) (34). The same inoculum was used to conduct the double synergy test, the double synergy test with disc matching, and the ATB^TM^ UR EU gallery test (08). The double synergy test is a widely used phenotypic reference method for detecting ESBL in Enterobacteriaceae. The double synergy test consists of looking for a synergistic image between a disc of amoxicillin + clavulanic acid (AMC 20/10 μg) and a disc of C3G [ceftazidime (CAZ) 30 μg and/or cefotaxime (CTX) 30 μg] and/or C4G [cefepime (FEP) 30 μg] and/or a monobactam [aztreonam (ATM) 30 μg]. An ESBL-producing Enterobacteriaceae is identified on the ATB UR EU (08) gallery when wells with Cefoxitin 32 (CX32) are susceptible (S) and those with CTX (and CAZ) are resistant (R).

### 2.5. Phenotypic confirmation tests for ESBL positive isolates

For the synergy tests, a distance of 30 mm was maintained between the discs' centers. The presence of ESBL is indicated by the presence of a “champagne cork” synergy image (Supplementary Figures 1, 2). Strains that failed the double synergy test were tested by bringing the C3G, C4G, and/or aztreonam discs (20 mm) closer to the MAC disc. Due to the possibility of co-production of a high level of cephalosporinase (cases), we used cloxacillin, a cephalosporinase inhibitor, to detect ESBL production. Cloxacillin is an antibiotic that inhibits the growth of bacteria in Enterobacteriaceae groups 1 and 2 at 0.25 mg/ml and group 3 at 0.3 mg/ml. However, it is ineffective against Gram-negative bacilli that produce penicillinases. The synergy test on Mueller-Hinton agar (MH) with cloxacillin is interpreted by comparing it to the synergy test without cloxacillin. The appearance of one or more synergistic images is considered positive. The restoration of inhibition diameters around cephalosporins indicates the presence of a possibly high level of cephalosporinase associated with ESBL.

### 2.6. Epidemiological and statistical data analysis

We created a database using the Sphinx Plus^2^—Lexica-V5 software, where we inserted all data for statistical analysis. The data was also analyzed with the R Statistical Software version 4.2.2 (R Core Team, 2021, R Foundation for Statistical Computing) in the tidyverse ecosystem. All variables were examined by univariate analysis using the χ^2^ or the Mann–Whitney *U*-tests as appropriate. All statistical tests were two-tailed, and a *p* < 0.05 was considered statistically significant.

### 2.7. Ethical considerations

The Ethics Committee of the University of Ngaoundere approved this study (Ref: N°2018/060/UN/R/RFS/CD-DSBM). The CPCAG authority reviewed and approved the study protocol. The confidentiality of the study subjects' information was maintained in accordance with national and international regulations.

## 3. Results

### 3.1. Isolation frequencies of *Enterobacteriaceae* associated with UTIs

**Figure 1:**
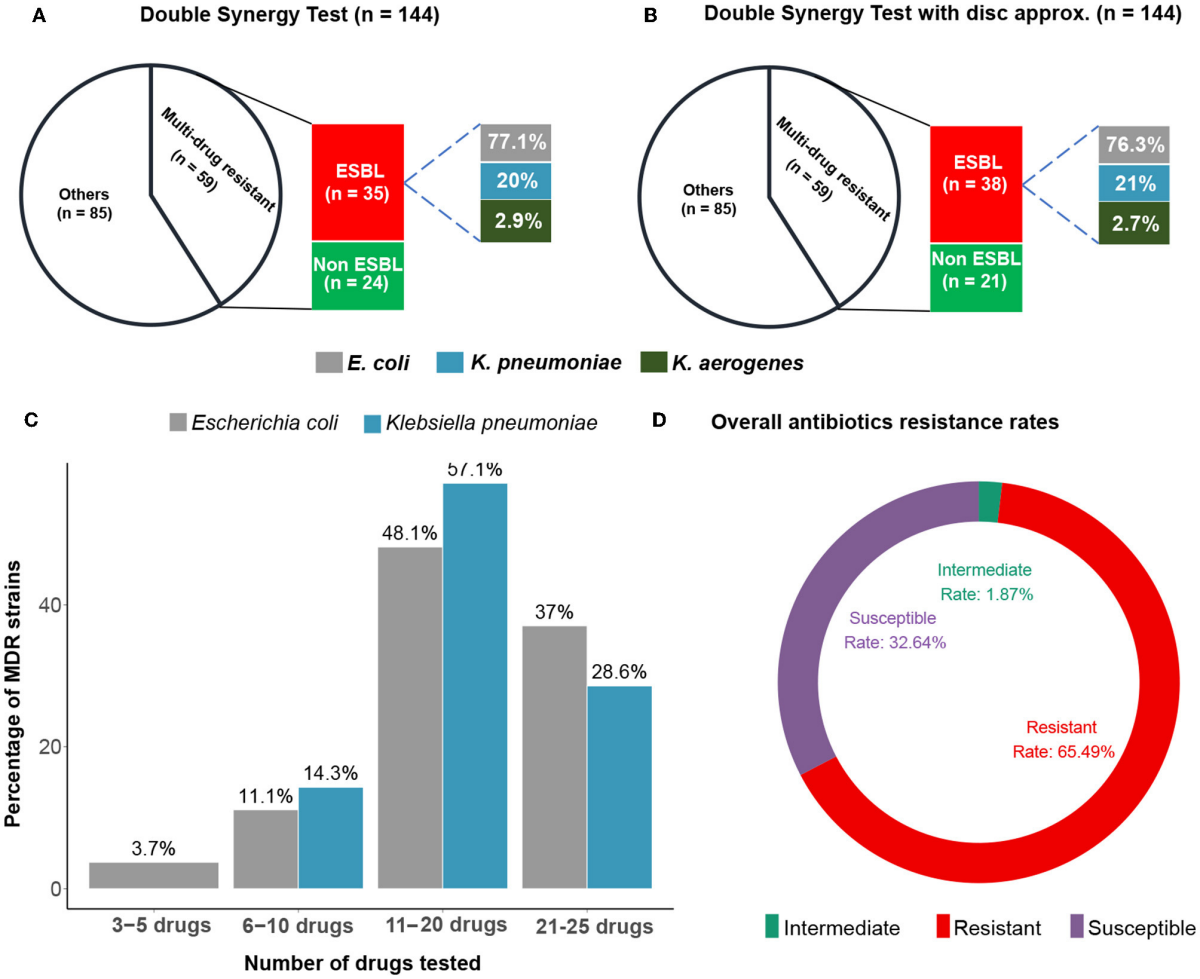
Double synergy tests. Number of isolates showing multi-drug co-resistance (MDR) and producing ESBL in the standard Double Synergy Test **(A)** and with disc approximation **(B)**. The percentages of MDR strains to different numbers of antibiotics **(C)**. Overall antibiotics resistance and susceptibility rates recorded among all isolates and drug tested **(D)**.

We isolated and identified 144 strains of Enterobacteriaceae from suspected UTIs cases. These included *Escherichia coli, Klebsiella pneumoniae, Enterobacter aerogenes, Enterobacter cloaceae, Citrobacter koseri, Proteus mirabilis*, and *Moeganella morganii*. *E. coli* (72.9%; 105/144) and *Klebsiella pneumoniae* (20.1%; 29/144) were by far the most frequently isolated strains, accounting for 93% of all isolates. The isolation rate for Enterobacter spp. was 3.5%, while other isolates accounted for less than 4% of the cases. Out of the 144 Enterobacteriaceae isolates, 59 (41%) were multi-resistant to at least three antibiotic families, with *E. coli* (76%) being by far the most abundant MDR strain, followed by *K. pneumoniae* (21%) (Figures 1A, B). Furthermore, 37% of *E. coli* MDR isolates and 28.6% of *K. pneumoniae* MDR isolates were co-resistant to more than 20 antibiotics, indicating a dramatic resistance rate for commonly used antibiotics (Figure 1C). Overall, around two-thirds (65.49%) of the UTIs isolates were tested positive for resistance, while roughly one-third (32.64%) was susceptible to all antibiotics (Figure 1D).

### 3.2. ESBL detection through phenotypic tests

The ATB UR EU (08) showed that 33 out of 59 MDR strains of Enterobacteriaceae were ESBL producers, i.e., a rate of 22.9% (33/144). The double synergy test showed that 35 of 59 MDR Enterobacteriaceae strains were ESBL producers at a rate of 24.3% (35/144). When only ESBL-producing strains were considered, *E. coli* was the most common ESBL+ at 77.1% (27/35), followed by *K. pneumoniae* at 20% (7/35) and *E. aerogenes* at 2.9% of the 35 detected ESBL-producing isolates (Figures 1A, B and Table 1). The disc-matching method identified 38 ESBL-producing strains compared to the double synergy test without matching, giving a total ESBL isolation rate of 26.38% (38/144). The results of the disc matching show that the other two methods failed detecting three ESBL positive strains (Table 1).

**Table 1:** Comparison of different phenotypic methods for ESBL detection.

**Number of strains tested (*n* = 59)**	**Number of ESBL+ strains per test category**		
	**Gallery screening test ATB UR EU (08)**	**Standard double Synergy test**	**Double Synergy test with disc appro-ximation**
*E. coli, n* = 48	27	27	29
*K. pneumoniae, n* = 10	5	7	8
*E. aerogenes, n* = 1	1	1	1
Total	33	35	38

### 3.3. Antibiotics resistance and susceptibility of ESBL-producing strains

**Figure 2:**
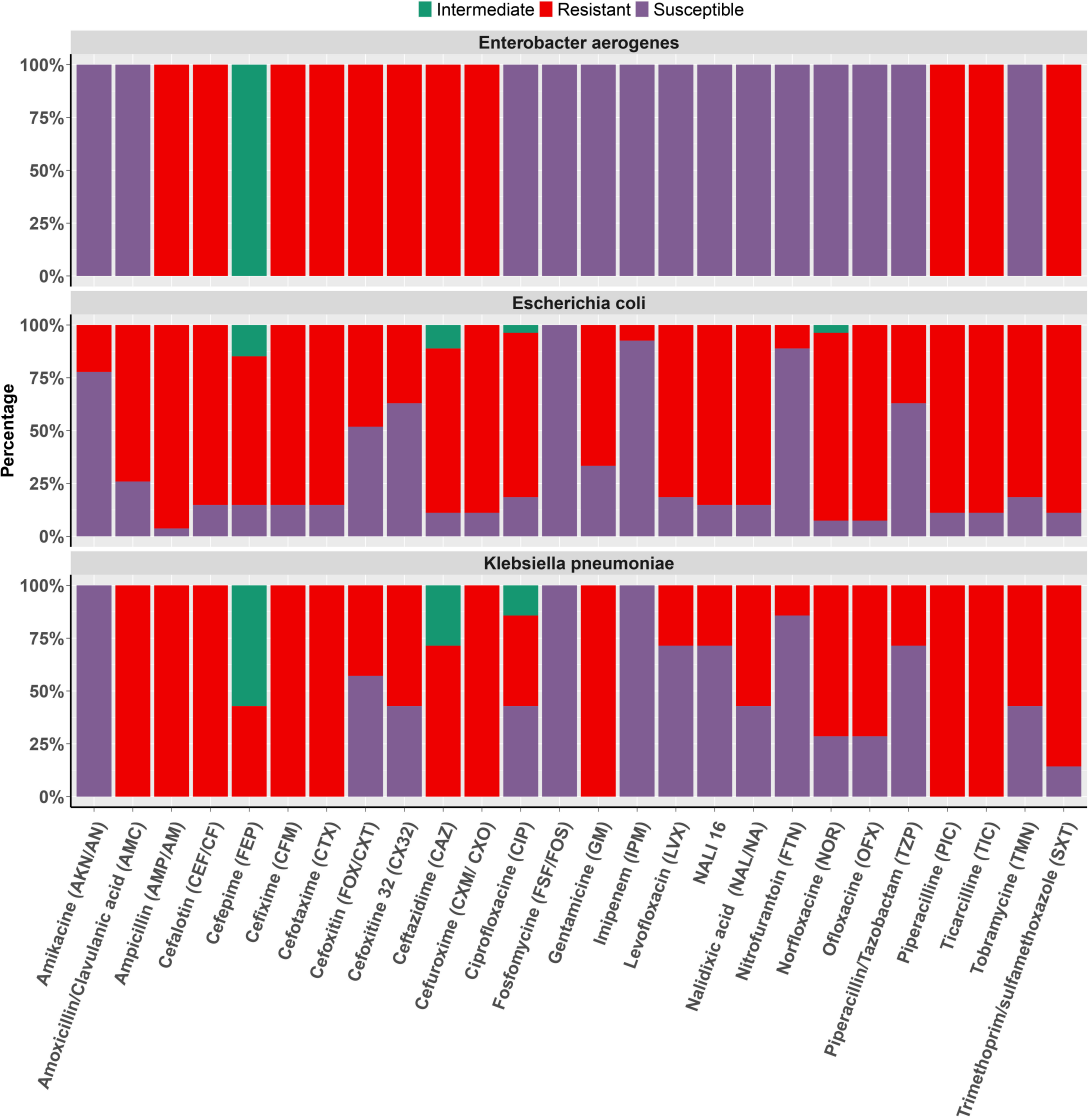
Antibiotic susceptibility. Co-resistance profile of ESBL+ isolates including *Escherichia coli, Klebsiella pneumoniae*, and *Enterobacter aerogenes* to the different antibiotics tested. Barplot depict percentage of Resistance, Susceptibility, and Intermediate strains for various commonly used antibiotics in Cameroon.

The overall resistance and susceptibility of MDR isolates, including *Escherichia coli, Klebsiella pneumoniae*, and *Enterobacter aerogenes*, to various antibiotics were assessed in this study. Our results suggested consistently higher resistance rates to most of the commonly used extended-spectrum antibiotics (Figure 2). The highest resistance rates were observed for ampicillin (98.31%), ticarcillin (94.92%), and piperacillin (91.53%), indicating that these drugs may no longer be effective in treating UTIs caused by these bacterial strains (Table 2). A substantial number of isolates also showed high levels of resistance to trimethoprim/sulfamethoxazole (86.44%), cephalothin (84.75%), and cefuroxime (77.97%). However, some antibiotics such as amoxicillin/clavulanic acid, nalidixic acid, and cefixime showed moderate effectiveness with resistance rates of 69.49, 71.19, and 64.41%, respectively.

**Table 2:** Overall resistance and susceptibility of multidrug-resistant isolates including *Escherichia coli, Klebsiella pneumonia* and *Enterobacter aerogenes*. R, resistant; S, susceptible; I, intermediate.

**Antibiotics**	**R (%)**	**S (%)**	**I (%)**
Ampicillin (AMP/AM)	98.31	1.69	0
Ticarcillin (TIC)	94.92	5.08	0
Piperacillin (PIC)	91.53	8.47	0
Trimethoprim/sulfamethoxazole (SXT)	86.44	13.56	0
Cephalothin (CEF/CF)	84.75	15.25	0
Cefuroxime (CXM/CXO)	77.97	22.03	0
Ofloxacin (OFX)	74.58	25.42	0
Norfloxacin (NOR)	72.88	25.42	1.69
Nalidixic acid (NAL/NA)	71.19	28.81	0
Amoxicillin/clavulanic acid (AMC)	69.49	30.51	0
NAL16	67.8	32.2	0
Cefixime (CFM)	64.41	35.59	0
Cefotaxime (CTX)	64.41	35.59	0
Ciprofloxacin (CIP)	59.32	37.29	3.39
Levofloxacin (LVX)	59.32	40.68	0
Tobramycin (TMN)	59.32	40.68	0
Ceftazidime (CAZ)	55.93	33.9	10.17
Gentamicin (GM)	54.24	45.76	0
Cefepime (FEP)	47.46	33.9	18.64
Cefoxitin (FOX/CXT)	37.29	62.71	0
Cefoxitin 32 (CX32)	33.9	66.1	0
Piperacillin/tazobactam (TZP)	33.9	66.1	0
Amikacin (AKN/AN)	13.56	86.44	0
Nitrofurantoin (FTN)	11.86	88.14	0
Imipenem (IPM)	5.08	94.92	0
Fosfomycin (FSF/FOS)	0	100	0

Antibiotics such as imipenem (94.92%), nitrofurantoin (88.14%), and amikacin (86.44%) were found to be relatively effective against the large majority of MDR strains. However, these last-resort antibiotics are typically reserved for severe infections, and their use may lead to the emergence of further resistance (Table 2). Intermediate susceptibility was observed only for norfloxacin, ciprofloxacin, and ceftazidime, with respectively 1.69, 3.39, and 10.17% of the isolates showing intermediate susceptibility to at least one antibiotic (Table 2). Regarding the prevalences of AMR among ESBL producers, the strains shared a high resistance rate to all beta-lactams, aminoglycosides, and fluoroquinolones/quinolones tested, with the exception of piperacillin/tazobactam (42.9%) and cefoxitin (42.9%), where they recorded a moderate coresistance rate. On the other hand, these strains also demonstrated low resistance to imipenem (5.70%), and fosfomycin was the only drug 100% effective against all ESBL strains tested (Figure 2).

### 3.4. Age and sex-specific antibiotics susceptibility

We investigated the proportion of multi-drug resistant ESBL+ isolates across gender and different age groups. Our data showed that males had a significantly higher proportion of MDR isolates compared to females (Mann–Whitney *U* test, *p* < 0.05), with a concerning ESBL producers rate of 43%. Male subjects have nearly twice as many ESBL+ isolates as females (Table 3). Regarding age groups, we observed the highest proportion (61%) of MDR isolates among children aged 0–12 years, with a relatively high ESBL+ prevalence of 41%. Interestingly, the 20–49 age group had the lowest proportion of MDR isolates, with a significantly lower ESBL+ rate of 7% (Mann–Whitney *U*-test, *p* < 0.05). Among individuals aged 50 years and above, the proportion of MDR isolates was intermediate (25%), with an ESBL+ rate of 17% (Table 3). These findings underscore the fact that the spread of ESBL+ is more pronounced in vulnerable populations such as children and older adults but also that UTIs in females are substantially less associated with MDR. This suggests age and sex-specific antibiotic susceptibility as potential risk factors for multi-drug resistance.

**Table 3:** Proportion of multi-drug resistant isolates along with the ESBL+ rate between genders and age groups.

		**Frequency MDR isolates (%)**	**EBSL+ rate (%)**
Gender	Males (*n* = 39)	66	43
	Females (*n* = 20)	34	22
Age group	0–12 years (*n* = 36)	61	41
	20–49 years (*n* = 8)	14	7
	50+ years (*n* = 15)	25	17

**Figure 3:**
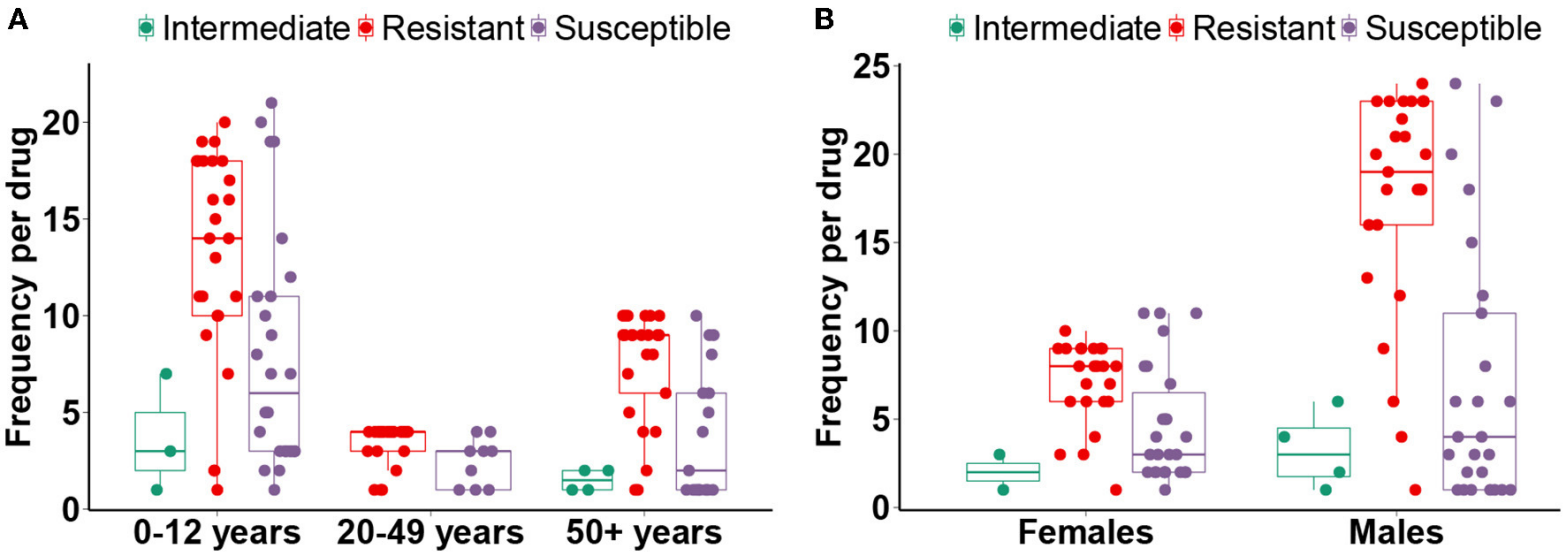
Age and sex-specific multi-drug resistance profiles. The distributions displaying the number of drugs to which MDR EBSL+ isolates are resistant, susceptible, or have intermediate susceptibility. The statistical significance tests were performed between age groups **(A)** and sexes **(B)**.

To better understand the age-and sex-specificity of the drug resistance patterns of MDR isolates, we assessed the number of drugs that the isolates were resistant to, susceptible to, or had intermediate susceptibility, in each age and sex group. The statistical analysis (Mann–Whitney *U*-test) showed a significantly high number of ineffective drugs among men vs. women isolates. Subjects aged 0–12 years exhibited the highest frequency of antibiotic coresistance, as well as the highest susceptibility. However, despite this relatively high drug susceptibility rate, only 31% of the MDR ESBL+ strains in this age group were susceptible to more than three antibiotics (Figure 3).

## 4. Discussion

### 4.1. Comparison of ATB UR EU (08) Gallery and Double Synergy Test

Effective detection of ESBL-producing bacteria is critical for making informed treatment decisions. In this study, we compared the performance of the ATB UR EU (08) Gallery and the double synergy test in detecting ESBL strains causing UTIs. Our results showed that the ATB UR EU (08) Gallery detected 33 ESBL strains, while the double synergy test detected 35 and 38 strains in close proximity. However, the ATB UR EU (08) Gallery could not detect three strains that were ESBL and cephalosporinase producers, showing its limitation in detecting strains with both characteristics. To confirm the presence of these strains, we used the synergy test with the discs brought together at a distance of 20 mm, which successfully detected all three strains. It should be noted that the ATB UR EU (08) Gallery did not report any false positives (strains identified as ESBL+ by the gallery but not confirmed by the synergy tests), indicating its overall reliability in detecting ESBL enterobacteria. Our findings suggest that medical laboratories using the ATB UR EU (08) Gallery can report the results of ESBL enterobacteria detected with this test with confidence. When a cephalosporin hyper-producing strain is suspected (positive when: CX32: R; CAZ: R and CTX: R), an optimized synergy test with disc reconciliation or the double disc test should be performed for more accurate detection. Our results agree with those of Garrec et al., who reported that reducing the distance between the discs improves the sensitivity of the double synergy test and that the sensitivity reaches 100% (35). Similarly, Tzelepi et al. pointed out that the sensitivity of this test increases from 61 to 90% by reducing the distance from 30 to 20 mm (36). Overall, we demonstrate that incorporating the ATB UR EU (08) Gallery and the synergy test can effectively monitor and control the spread of ESBL+ antimicrobial resistance, particularly in resource-constrained settings.

### 4.2. Prevalence and susceptibility of ESBL producers to the different antibiotics

There has been a worldwide growing concern about the prevalence of ESBL producing bacteria and their association with urinary tract infections (37, 38). This issue is problematic in Cameroon, given the country's high burden of infectious diseases and the limited availability of effective antibiotics (39). Several studies have previously investigated the prevalence of ESBL-associated UTIs in Cameroon. One such study by (20) reported an ESBL-E prevalence rate of 34.4% in children aged 0–2 years affected by blood stream infections. Our study reports an overall ESBL-associated UTIs prevalence rate of 26.3% (38 out of 144), which is in the range of previous studies (19.3 to 48.2%) (19, 20). This rate is however lower than the ESBL+ prevalence reported in other African countries such as Algeria (37.1%) (40) and Tunisia (30.8%) (41) but higher than the rate reported in Morocco (12.2%) (42). The majority of patients in our study population had a history of failed antibiotic therapy, prior antibiotic use, and recent hospitalization, which could explain the relatively high prevalence of multi-drug resistant ESBL+ isolates. A common feature of the bacterial isolates tested in this study is their high co-resistance (pan-resistance) to all beta-lactams tested, with the exception of piperacillin/tazobactam (TZP) and cefoxitin (FOX/CXT), which showed a ESBL+ sensitivity of 42.9 and 57.1%, respectively.

It is also concerning that even last-line antibiotics like imipenem (carbapenem) were not sensitive against nearly 6% of the ESBL+ strains, revealing an emerging resistance against this last resort antimicrobial drug. This is alarming because similar co-resistance rates, except for Piperacillin/Tazobactam, have been reported in other regions in Cameroon (2, 19), showing a growing national or even a global trend. This situation may be the result of selection pressure caused by the overprescription and sometimes abusive use of broad-spectrum antibiotics in hospitals and the community, including non-prescription dispensing and self-medication. Furthermore, environmental residual antibiotics can exert selective pressure on bacteria. AMR has been linked to human-caused antibiotic contamination in the environment, such as the discharge of treated wastewater and the spread of sludge from sewage treatment plants and livestock effluents (43). Numerous antibiotics, including nitroimidazoles, sulfonamides, macrolides, and trimethoprim, have been found in industrial livestock effluents or fish farms (44). Guillon et al. discovered danofloxacin, an antibiotic used in veterinary medicine, in very low doses in certain drinking water (45).

The development of carbapenems (imipenem) resistance, which are actually susceptible to ESBL+ strains, may also drive the emergence of novel strains combining both ESBL expression and carbapenem impermeability. Co-expression of ESBL and carbapenemases can lead to a “pan-resistance” phenotype to beta-lactams (46). The percentages of ESBL+ resistance to gentamicin (61%) and tobramycin (52.2%) are higher than to amikacin (31.4%), confirming that the latter is still the most effective aminoglycoside against ESBL producers, as reported in several studies (2, 19, 31). Our finding also highlighted a resistance to all quinolones and fluoroquinolones tested. The high rate of ESBL+ co-resistance to these antibiotics, particularly ciprofloxacin, points out to its decreased effectiveness in the treatment of urinary tract infections (47). This is primarily explained by the widespread use of ciprofloxacin and norfloxacin as the first-line treatment of UTIs due to their broad bacterial spectra and sound urine diffusion (48).

This study raises the threat of complete impasse of antimicrobial therapy as many first-line antibiotics showed low sensitivity to ESBL-associated UTIs. The high prevalence of ESBL in UTIs in Cameroon may be attributed to a number of factors, including the inappropriate use of broad-spectrum antibiotics in hospitals and the community, inadequate infection control measures in healthcare settings, and limited access to laboratory testing for accurate diagnosis and treatment. This significant ESBL+ co-resistance severely limits the therapeutic arsenal and increases the risk of treatment failure (31). However, fosfomycin was completely effective against ESBL+ strains. Fosfomycin retains good activity against all isolates tested, which is most likely due to its low prescription, as it is recommended for treating acute and critical cases. In Cameroon and other Sub-Saharan African countries, the problem is the high cost of fosfomycin, which is frequently out of reach for the population, as well as its scarcity in pharmacies (19, 30). Given these insights, it is critical to implement strategies to reduce the prevalence and spread of ESBL-producing bacteria. This includes promoting antibiotic prudence, improving infection control measures in healthcare settings, and expanding access to laboratory testing for accurate diagnosis and treatment.

### 4.3. Epidemiological risk factors

Our study indicate that there are significant differences in the proportion of multi-drug resistant isolates with ESBL positive rate between different gender and age groups. ESBL-associated UTIs were more common in males than in females subjects, with a male/female sex ratio of 2. Additionally, the 0-12 age group had the highest baseline prevalence of UTIs and the highest rate of MDR isolates and ESBL-positive rates, while the 20–49 age group had the lowest. These findings are in line with previous studies that have reported higher rates of ESBL+ and MDR isolates in males and in children (1, 49). The reasons for these differences are not clearly understood but could include differential exposure to beta-lactam and broad-spectrum antibiotics, differences in infection control practices, and cultural and genetic factors (50). For example, circumcision may be more likely to increase the susceptibility of males subject to UTIs (51), and thus the exposure to antibiotic-resistant strains including ESBL-producers. At the same time, children may be more susceptible to UTIs because of their immune vulnerability (49). A limitation of our study is the small sample size for some age groups, which may limit the generalizability of our findings. Furthermore, our study did not investigate the mechanisms underlying the differences in MDR and ESBL+ rates between gender and age groups. Thus, more research is needed to clarify these probable risk factors for ESBL-associated MDR in urinary tract infections.

### 4.4. Outlook and future analyses

The current study has focused on the phenotypic characterization and prevalence of MDR ESBL-producing strains associated with UTIs in a clinical context, by providing relevant data for AMR clinical stewardship. However, a future direction would be the use of next-generation sequencing (NGS) for genotyping the identified ESBL-producing isolates to gain deeper insights into the molecular mechanisms underlying their multidrug resistance. For example, whole-genome sequencing of these isolates panels could help to identify genetic determinants of ESBL+ resistance, such as mobile genetic elements including plasmids and integrons. In addition, genotyping of ESBL-producing bacteria could provide crucial information on the genomic epidemiology and population structure of these pathogens, which would then inform the development of public health guidelines and surveillance measures for MDR-ESBL variants.

## 5. Conclusions

In conclusion, the multi-drug resistant isolates in this study showed high resistance levels to the most commonly used antibiotics in Cameroon. Our study provides evidence for significant differences in the proportion of MDR isolates with the ESBL positive rate between different gender and age groups. These findings underscore the importance of continued surveillance and the development of effective strategies to prevent the spread or the emergence of antibiotic-resistant strains in the population. The high isolation frequency of ESBL+ strains requires clinicians to prescribe antibiotics rationally to reduce the selection pressure exerted by broad-spectrum antibiotic therapy. Antibiotics with a low co-resistance rate, such as amikacin, may be an alternative treatment. For the epidemiological surveillance of MDR, detecting enterobacteria BLSE+ strains should be a routine examination in the suspicion of enterobacteria infections. Based on our results, the ATB^TM^ UR EU (08) gallery can effectively screen ESBL+ strains for diagnostics, avoiding additional costs to the patient and ensuring a better patient management and AMR surveillance in our laboratories.

## Data availability statement

The original contributions presented in the study are included in the article/Supplementary material, further inquiries can be directed to the corresponding authors.

## Ethics statement

The studies involving human participants were reviewed and approved by the Ethics Committee of the University of Ngaoundere approved this study (Ref: N°2018/060/UN/R/RFS/CD- DSBM). The CPCAG authority reviewed and approved the study protocol. The confidentiality of the study subjects' information was maintained in accordance with national and international regulations. Written informed consent to participate in this study was provided by the participants' legal guardian/next of kin.

## Author contributions

KD-A-N, BM, LO, HM, LD, NY, and JN: conceptualization, methodology, and software. BM, SF, and NY: supervision. KD-A-N, BM, LO, HM, LD, and NY: data collection—curation. KD-A-N, LO, NY, and JN: resources and formal analysis. KD-A-N and JN: funding acquisition and writing—original draft preparation. KD-A-N, BM, LO, HM, CT, EE, MM, SF, LD, NY, and JN: writing—review and editing. All authors contributed to the article and approved the submitted version.

## References

[1] Pitout JD Nordmann P Laupland KB Poirel L. Emergence of Enterobacteriaceae producing extended-spectrum *β*-lactamases (ESBLs) in the community. J Antimicrob Chemother. (2005) 56:52–9. 10.1093/jac/dki166 15917288

[2] Sbiti M Lahmadi K Louzi L. Profil épidémiologique des entérobactéries uropathogènes productrices de bêta-lactamases à spectre élargi. Pan Afr Med J. (2017) 28:1–9. 10.11604/pamj.2017.28.29.11402 PMC5681015 29138665

[3] Bradford PA. Extended-spectrum *β*-lactamases in the 21st century: characterization, epidemiology, and detection of this important resistance threat. Clin Microbiol Rev. (2001) 14:933–51. 10.1128/CMR.14.4.933-951.2001 11585791 PMC89009

[4] Pana ZD Zaoutis T. Treatment of extended-spectrum *β*-lactamase-producing Enterobacteriaceae (ESBLs) infections: what have we learned until now? F1000Res. (2018) 7. 10.12688/f1000research.14822.1 30228863 PMC6117850

[5] Bassetti M Merelli M Temperoni C Astilean A. New antibiotics for bad bugs: where are we? Ann Clin Microbiol Antimicrob. (2013) 12:1–15. 10.1186/1476-0711-12-22 23984642 PMC3846448

[6] Chakraborty D Basu S Das S. A study on infections caused by metallo beta lactamase producing gram negative bacteria in intensive care unit patients. Am J Infect Dis. (2010) 6:34–9. 10.3844/ajidsp.2010.34.39

[7] D'Andrea MM Arena F Pallecchi L Rossolini GM. CTX-M-type *β*-lactamases: a successful story of antibiotic resistance. Int J Med Microbiol. (2013) 303:305–17. 10.1016/j.ijmm.2013.02.008 23490927

[8] WHO. Prioritization of Pathogens to Guide Discovery, Research and Development of New Antibiotics for Drug-Resistant Bacterial Infections, Including Tuberculosis. Geneva: WHO Press (2017). Available online at: https://www.who.int/publications/i/item/WHO-EMP-IAU-2017.12 (accessed January 30, 2023).

[9] Park JJ Seo YB Lee J. Antimicrobial susceptibilities of Enterobacteriaceae in community-acquired urinary tract infections during a 5-year period: a single hospital study in Korea. Infect Chemother. (2017) 49:184–93. 10.3947/ic.2017.49.3.184 29027385 PMC5620385

[10] Lagunas-Rangel FA. Antimicrobial susceptibility profiles of bacteria causing urinary tract infections in Mexico: single-centre experience with 10 years of results. J Glob Antimicrob Resist. (2018) 14:90–4. 10.1016/j.jgar.2018.03.004 29581074

[11] Raphael E Glymour MM Chambers HF. Trends in prevalence of extended-spectrum beta-lactamase-producing *Escherichia coli* isolated from patients with community-and healthcare-associated bacteriuria: results from 2014 to 2020 in an urban safety-net healthcare system. Antimicrob Resist Infect Control. (2021) 10:1–13. 10.1186/s13756-021-00983-y 34380549 PMC8359060

[12] Woerther PL Burdet C Chachaty E Andremont A. Trends in human fecal carriage of extended-spectrum *β*-lactamases in the community: toward the globalization of CTX-M. Clin Microbiol Rev. (2013) 26:744–58. 10.1128/CMR.00023-13 24092853 PMC3811232

[13] Kassakian SZ Mermel LA. Changing epidemiology of infections due to extended spectrum beta-lactamase producing bacteria. Antimicrob Resist Infect Control. (2014) 3:1–6. 10.1186/2047-2994-3-9 PMC4230027 24666610

[14] Thaden JT Fowler VG Sexton DJ Anderson DJ. Increasing incidence of extended-spectrum *β*-lactamase-producing *Escherichia coli* in community hospitals throughout the southeastern United States. Infect Control Hosp Epidemiol. (2016) 37:49–54. 10.1017/ice.2015.239 26458226 PMC4748740

[15] El Bouamri M Arsalane L Kamouni Y Berraha M Zouhair S. Évolution récente du profil épidémiologique des entérobactéries uropathogènes productrices de *β*-lactamases à spectre élargi à Marrakech, Maroc. Progrès en urologie. (2014) 24:451–5. 10.1016/j.purol.2013.11.010 24861685

[16] Meng M Li Y Yao H. Plasmid-mediated transfer of antibiotic resistance genes in soil. Antibiotics. (2022) 11:525. 10.3390/antibiotics11040525 35453275 PMC9024699

[17] Lerminiaux NA Cameron AD. Horizontal transfer of antibiotic resistance genes in clinical environments. Can J Microbiol. (2019) 65:34–44. 10.1139/cjm-2018-0275 30248271

[18] Thangaraju P Venkatesan S. WHO Ten threats to global health in 2019: antimicrobial resistance. Cukurova Med J. (2019) 44:1150–1. 10.17826/cumj.514157

[19] Djim-Adjim-Ngana K Oumar LA Mbiakop BW Njifon HLM Crucitti T Nchiwan EN. Prevalence of extended-spectrum beta-lactamase-producing enterobacterial urinary infections and associated risk factors in small children of Garoua, Northern Cameroon. Pan Afr Med J. (2020) 36. 10.11604/pamj.2020.36.157.21347 PMC7436643 32874421

[20] Djuikoue CI Djouela Djoulako PD Wouambo RK Lacmago ST Dayomo A Kamga HG. Prevalence of *Escherichia coli* producing extended spectrum beta-lactamase (ESBL) driven septicaemia in children aged 0–2 years in two districts hospitals in Yaounde, Cameroon. Bacteria. (2022) 1:294–301. 10.3390/bacteria1040022

[21] Gangoué-Piéboji J Bedenic B Koulla-Shiro S Randegger C Adiogo D Ngassam P. Extended-spectrum-*β*-lactamase-producing Enterobacteriaceae in Yaounde, Cameroon. J Clin Microbiol. (2005) 43:3273–7. 10.1128/JCM.43.7.3273-3277.2005 16000447 PMC1169189

[22] Lonchel CM Meex C Gangoué-Piéboji J Boreux R Assoumou MCO Melin P. Proportion of extended-spectrum ß-lactamase-producing Enterobacteriaceae in community setting in Ngaoundere, Cameroon. BMC Infect Dis. (2012) 12:53. 10.1186/1471-2334-12-53 22405322 PMC3329637

[23] Lonchel CM Melin P Gangoueé-Pieéboji J Assoumou MCO Boreux R De Mol P. Extended-spectrum *β*-lactamase-producing Enterobacteriaceae in Cameroonian hospitals. Eur J Clin Microbiol Infect Dis. (2013) 32:79–87. 10.1007/s10096-012-1717-4 22886058

[24] Magoué CL Melin P Gangoué-Piéboji J Assoumou MCO Boreux R De Mol P. Prevalence and spread of extended-spectrum *β*-lactamase-producing Enterobacteriaceae in Ngaoundere, Cameroon. Clin Microbiol Infect. (2013) 19:E416–20. 10.1111/1469-0691.12239 23647948

[25] Shah A Hasan F Ahmed S Hameed A. Extended-spectrum *β*-lactamases (ESBLs): characterization, epidemiology and detection. Crit Rev Microbiol. (2004) 30:25–32. 10.1080/10408410490266429 15116761

[26] Yousefipour M Rasoulinejad M Hadadi A Esmailpour N Abdollahi A Jafari S. Bacteria producing extended spectrum *β*-lactamases (ESBLs) in hospitalized patients: prevalence, antimicrobial resistance pattern and its main determinants. Iran J Pathol. (2019) 14:61. 10.30699/ijp.14.1.61 31531102 PMC6708561

[27] Djuikoue IC Woerther PL Toukam M Burdet C Ruppé E Gonsu KH. Intestinal carriage of Extended Spectrum Beta-Lactamase producing E. coli in women with urinary tract infections, Cameroon. J Infect Dev Count. (2016) 10:1135–9. 10.3855/jidc.7616 27801378

[28] Djuikoue I Njajou O Kamga H Fokunang C Bongoe A Bruno E. Prévalence des?-lactamases CTX-M chez *Escherichia coli* provenant d'infections urinaires acquises dans la communauté et facteurs de risque associés chez les femmes au Cameroun. J Epidemiol Res. (2017) 3:51. 10.5430/jer.v3n1p51

[29] Masterton R Drusano G Paterson D Park G. Appropriate antimicrobial treatment in nosocomial infections–the clinical challenges. J Hosp Infect. (2003) 55:1–12. 10.1016/S0195-6701(03)00294-9 14623198

[30] Ndir A Diop A Faye PM Cissé MF Ndoye B Astagneau P. Epidemiology and burden of bloodstream infections caused by extended-spectrum beta-lactamase producing Enterobacteriaceae in a pediatric hospital in Senegal. PLoS ONE. (2016) 11:e0143729. 10.1371/journal.pone.0143729 26867226 PMC4750952

[31] Toudji AG Djeri B Karou SD Tigossou S Ameyapoh Y De Souza C. Prévalence des souches d'entérobactéries productrices de bêta-lactamases à spectre élargi isolées au Togo et de leur sensibilité aux antibiotiques. Int J Biol Chem Sci. (2017) 11:1165–77. 10.4314/ijbcs.v11i3.19

[32] Surgers L Jolivot PA Lalande V Pacanowski J Homor A Boëlle PY. P-14: Épidémiologie clinique et bactériologique des BLSE et facteurs de risque de mortalité associés aux infections. Méd Maladies Infect. (2014) 44:85. 10.1016/S0399-077X(14)70303-8

[33] Lustiner France. Galerie ATB^*TM*^ *UR EU [Antibiogramme / Norme NCCLS] Biomérieux* (2019). Available online at: https://www.lustiner.com/Articles/3985917/Galerie-ATB-8482-UR-EU-Antibiogramme-Norme-NCCLS-Biomerieux-/search%5Bpos%5D=40&search%5Btags%5D=273 (accessed January 30, 2023).

[34] Richard B, Jean-Pierre, B, François, C, Vincent, C, Patrice, C, Luc, D,. Société Française de Microbiologie (2019). Available online at: https://www.sfm-microbiologie.org/2019/05/06/casfm-eucast-2019-v2/ (accessed January 30, 2023).

[35] Garrec H Drieux-Rouzet L Golmard JL Jarlier V Robert J. Comparison of nine phenotypic methods for detection of extended-spectrum *β*-lactamase production by Enterobacteriaceae. J Clin Microbiol. (2011) 49:1048–57. 10.1128/JCM.02130-10 21248086 PMC3067698

[36] Tzelepi E Giakkoupi P Sofianou D Loukova V Kemeroglou A Tsakris A. Detection of extended-spectrum *β*-lactamases in clinical isolates of *Enterobacter cloacae* and *Enterobacter aerogenes*. *J Clin Microbiol*. (2000) 38:542–6. 10.1128/JCM.38.2.542-546.2000 PMC86144 10655342

[37] Chiang CY Uzoma I Moore RT Gilbert M Duplantier AJ Panchal RG. Mitigating the impact of antibacterial drug resistance through host-directed therapies: current progress, outlook, and challenges. MBio. (2018) 9:e01932–17. 10.1128/mBio.01932-17 PMC5790911 29382729

[38] Saipriya J Shubha D Sudhindra K Sumantha A Madhuri K. Clinical importance of emerging ESKAPE pathogens and antimicrobial susceptibility profile from a tertiary care centre. Int J Curr Microbiol Appl Sci. (2018) 7:2881–91. 10.20546/ijcmas.2018.705.336

[39] Massongo M Ngando L Pefura Yone E NZouankeu A Mbanzouen W Fonkoua M. Trends of antibacterial resistance at the national reference laboratory in Cameroon: comparison of the situation between 2010 and 2017. BioMed Res Int. (2021) 2021:1–10. 10.1155/2021/9957112 34124266 PMC8166466

[40] Souna D Sefraoui I Drissi M. Résistance aux antibiotiques des entérobactéries au niveau du CHU de Sidi Bel Abbes (Algérie). Microbiol Hyg Alim. (2011) 23:37–41.

[41] Hammami S Saidani M Ferjeni S Aissa I Slim A Boutiba-Ben Boubaker I. Characterization of extended spectrum *β*-lactamase-producing *Escherichia coli* in community-acquired urinary tract infections in Tunisia. Microb Drug Resist. (2013) 19:231–6. 10.1089/mdr.2012.0172 23363379

[42] Amine IL Chegri M L'kassmi H. Épidémiologie et résistance aux antibiotiques des entérobactéries isolées d'infections urinaires à l'hôpital militaire Moulay-Ismail de Meknès. Antibiotiques. (2009) 11:90–6. 10.1016/j.antib.2008.10.004

[43] Tello A Austin B Telfer TC. Selective pressure of antibiotic pollution on bacteria of importance to public health. Environ Health Perspect. (2012) 120:1100–6. 10.1289/ehp.1104650 22571927 PMC3440082

[44] Haguenoer JM. Les résidus de médicaments présentent-ils un risque pour la santé publique? Santé Publique. (2010) 22:325–42. 10.3917/spub.103.0325 20858332

[45] Guillon A Noyon N Gogot C Robert S Bruchet A Esperanza M. Study on veterinary and human antibiotics in raw and treated water from a French basin. Water Sci Technol Water Supply. (2015) 15:1275–84. 10.2166/ws.2015.094

[46] Aminzadeh Z Sadat Kashi M Sha'bani M. Bacteriuria by extended-spectrum Beta-lactamase-producing Escherichia coli and Klebsiella pneumoniae: isolates in a governmental hospital in South of Tehran, Iran. Iran J Kidney Dis. (2008) 2:197–200. 19377237

[47] Gagliotti C Buttazzi R Sforza S Moro ML Group ERARS. Resistance to fluoroquinolones and treatment failure/short-term relapse of community-acquired urinary tract infections caused by *Escherichia coli*. *J Infect*. (2008) 57:179–84. 10.1016/j.jinf.2008.07.004 18707763

[48] Lee MTG Lee SH Chang SS Lee SH Lee M Fang CC. Comparative effectiveness of different oral antibiotics regimens for treatment of urinary tract infection in outpatients: an analysis of national representative claims database. Medicine. (2014) 93. 10.1097/MD.0000000000000304 25526477 PMC4603088

[49] Daniel M Szymanik-Grzelak H Sierdziński J Podsiadły E Kowalewska-Młot M Pańczyk-Tomaszewska M. Epidemiology and risk factors of UTIs in Children–a single-center observation. J Pers Med. (2023) 13:138. 10.3390/jpm13010138 36675799 PMC9865477

[50] Nasrollahian S Halaji M Hosseini A Teimourian M Armaki MT Rajabnia M. Genetic diversity, carbapenem resistance genes, and biofilm formation in UPEC isolated from patients with catheter-associated urinary tract infection in North of Iran. Int J Clin Pract. (2022) 2022. 10.1155/2022/9520362 PMC9507725 36187911

[51] Shaikh N Morone NE Bost JE Farrell MH. Prevalence of urinary tract infection in childhood: a meta-analysis. Pediatr Infect Dis J. (2008) 27:302–8. 10.1097/INF.0b013e31815e4122 18316994

