# Supplementary material for: Phenotypic characterization and epidemiology of extended-spectrum β-lactamase-producing Enterobacteriaceae strains from urinary tract infections in Garoua, Cameroon
Source: Front Public Health. 2023 Jun 29;11:1187934. doi: 10.3389/fpubh.2023.1187934 (PMC10343957; doi:10.3389/fpubh.2023.1187934)
Supplement: Supplementary file 2 [file Data_Sheet_2.docx]

**Supplementary Figures: Phenotypic characterization and epidemiology
of extended-spectrum β-lactamase-producing Enterobacteriaceae strains isolated from urinary tract infections in Garoua, Cameroon**


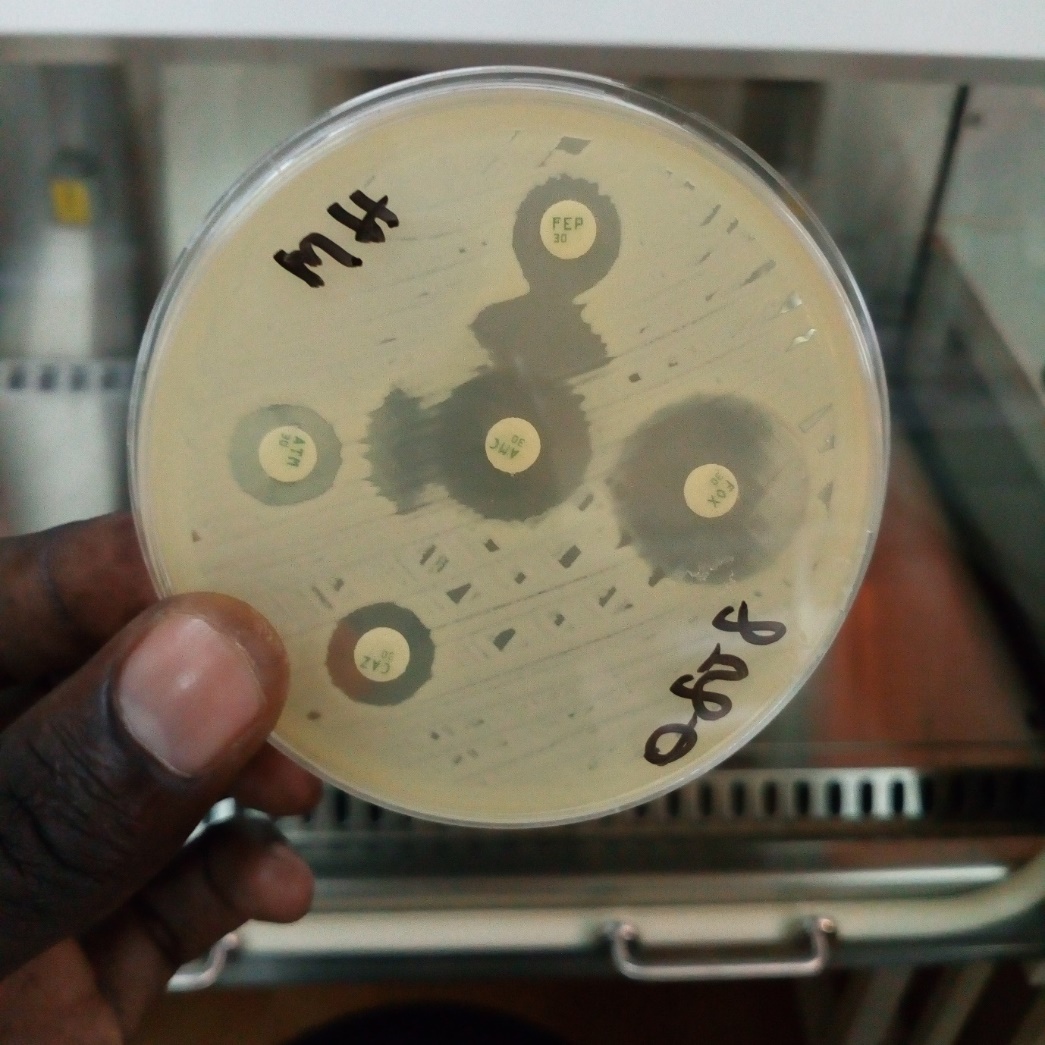


**Figure S1:** double disc synergy test (DDST) positive between amoxicillin/clavulanic acid (AMC) and cefepime (FEP) and aztreonam (ATM) (appearance in “champagne cap”).


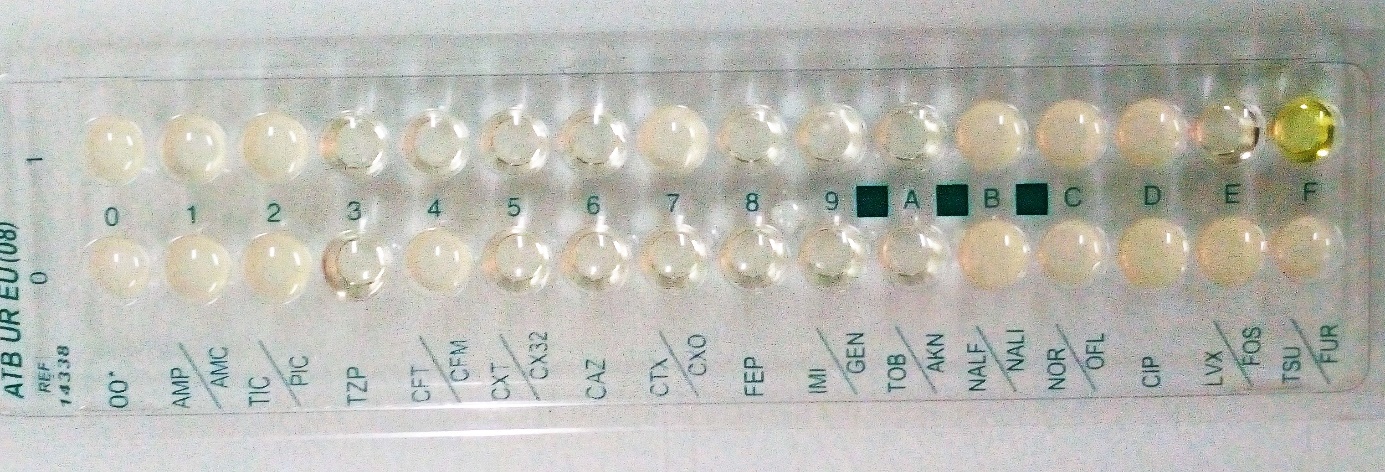


**Figure S2 :** Example of a strain of enterobacteria producing ESBL identified using the ATB UR EU gallery (08) (Biomerieux' SA, France) (Cefoxitin 32 (CX32) are susceptible (S) and those with CTX (and CAZ) are resistant (R))


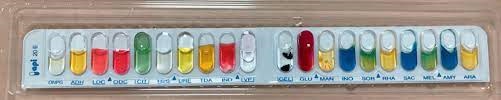


**Fig. S3: standard API kit for identification of enterobacteria**
